# In Vitro Evaluation of the Antioxidant, Cytoprotective, and Antimicrobial Properties of Essential Oil from *Pistacia vera* L. Variety Bronte Hull

**DOI:** 10.3390/ijms18061212

**Published:** 2017-06-06

**Authors:** Antonella Smeriglio, Marcella Denaro, Davide Barreca, Antonella Calderaro, Carlo Bisignano, Giovanna Ginestra, Ersilia Bellocco, Domenico Trombetta

**Affiliations:** Department of Chemical, Biological, Pharmaceutical and Environmental Sciences, University of Messina, Viale F. Stagno d’Alcontres 31, 98166 Messina, Italy; asmeriglio@unime.it (A.S.); denaromarcella.md@gmail.com (M.D.); anto.calderaro@gmail.com (A.C.); cbisignano@unime.it (C.B.); gginestra@unime.it (G.G.); ebellocco@unime.it (E.B.); dtrombetta@unime.it (D.T.)

**Keywords:** *Pistacia vera* L. variety Bronte, essential oil, antioxidant, cytoprotective activity, antimicrobial activity

## Abstract

Although the chemical composition and biological properties of some species of the genus *Pistacia* has been investigated, studies on hull essential oil of *Pistacia vera* L. variety Bronte (HEO) are currently lacking. In this work, we have carried out an in-depth phytochemical profile elucidation by Gas Chromatography-Mass Spectrometry (GC-MS) analysis, and an evaluation of antioxidant scavenging properties of HEO, using several different in vitro methods, checking also its cytoprotective potential on lymphocytes treated with tert-butyl hydroperoxide. Moreover, the antimicrobial activity against Gram-positive and Gram-negative strains, both American Type Culture Collection (ATCC) and clinical isolates, was also investigated. GC-MS analysis highlighted the richness of this complex matrix, with the identification of 40 derivatives. The major components identified were 4-Carene (31.743%), α-Pinene (23.584%), d-Limonene (8.002%), and 3-Carene (7.731%). The HEO showed a strong iron chelating activity and was found to be markedly active against hydroxyl radical, while scarce effects were found against 2,2-diphenyl-1-picrylhydrazyl (DPPH) radical. Moreover, pre-treatment with HEO was observed to significantly increase the cell viability, decreasing the lactate dehydrogenase (LDH) release. HEO was bactericidal against all the tested strains at the concentration of 7.11 mg/mL, with the exception of *Pseudomonas aeruginosa* ATCC 9027. The obtained results demonstrate the strong free-radical scavenging activity of HEO along with remarkable cytoprotective and antimicrobial properties.

## 1. Introduction

Plants producing essential oils represent a large part of natural flora and an important resource in various fields such as the pharmaceutical, food, and cosmetic industries, thanks to their flavour, fragrance, and biological activities [1]. Essential oils are a complex mixture of hydrocarbons and oxygenated hydrocarbons arising from the isoprenoid pathways and secreted by glandular trichomes disseminated mainly onto the surface of plant organs; therefore, they have a pivotal role in the growth and colonization of plants, conferring colour and scent to reproductive organs, attracting pollinators and favouring seed dispersal [2]. Furthermore, they seem to mediate the plant relationship with abiotic (e.g., light, temperature, and so on) and biotic factors, playing a defensive role against herbivores, harmful insects, and microbial pathogens [2].

More than 250 types of essential oils are commercialized annually on the international market, some of which are employed in aromatherapy and for the treatment of several diseases including cardiovascular and neurological diseases, diabetes, and cancer [1]. Moreover, the antimicrobial properties of essential oils have been widely recognized [3,4,5,6] thanks to several studies which showed a synergistic effect of several components of essential oils against various human pathogens [1]. Essential oils have been used since ancient times, in folk medicine throughout the world, to preserve a good health status [6,7,8,9]. Presently, several properties and therapeutic effects have been ascribed to essential oils such as antibiotic, rubefacient, anaesthetic, antispasmodic, balmy-expectorant, repellent, carminative, as well as beneficial effects on the central and peripheral nervous system [10,11,12,13,14,15].

Recently, antimicrobial drug resistance has brought researchers to evaluate novel antimicrobial lead molecules to treat various human pathogens. Synthetic drugs have often failed this goal, if not for a lack of efficacy, because of the greater risk to which the patient was exposed (acute and chronic toxicity and environmental hazard potential), leading the researchers to better explore natural remedies [1]. From this point of view, the study of essential oils has undergone a remarkable growth, and many new plants producing essential oil have been evaluated [16,17,18,19,20]. Among these, the authors decided to put their attention on *Pistacia vera* L., a nut tree that belongs to the Anacardiaceae family [21] cultivated in Iran, Turkey, the USA, Syria, Italy, Tunisia, and Greece.

In Italy, pistachio crops are typically Sicilian, mainly grown in the territory of Bronte (CT), situated on the eastern part of Sicily and characterized by lava-rich soils and very particular climatic conditions [21,22] which confer to the pistachio nuts superior organoleptic and nutritional characteristics. For this reason, the European Union recognized the variety Bronte as a D.O.P. (Protected Designation of Origin) product. Pistachio nuts are considered a rich source of many important biofunctional compounds that are useful for the human diet and known for their various pharmacological properties such as antimicrobial, anti-inflammatory, insecticidal, and anti-nociceptive activities [23]. Recently, we analysed and described the nutraceutical, antioxidant, and cytoprotective activity of crude phenols and anthocyanins-rich extracts derived from ripe pistachio hulls (*Pistacia vera* L., variety Bronte), a by-product of the pistachio industry [23,24]. These studies managed to identify this matrix as a promising source of healthy compounds. The results obtained highlighted antioxidant and cytoprotective properties directly correlated to the high total phenol content, in particular flavonols, phenolic acid, and flavan-3-ols and, among anthocyanins, to the high content of cyanidyn-3-*O*-galactoside [23,24]. The presence of these compounds makes pistachio hulls an attractive source of health-promoting compounds, which are potentially helpful against several oxidative stress-related diseases.

Despite the well-known ethnopharmacological relevance of some essential oils derived from some species of the genus *Pistacia* (*P. atlantica* Desf and *P. integerrima* J.L. Stewart ex Brandis) against several diseases, particularly those affecting the respiratory and the gastrointestinal tract [25,26,27,28], no literature data concerning the hull essential oil composition and biological properties of *Pistacia vera* L., variety Bronte, are today available.

Having considered the above, we have for the first time performed, a phytochemical characterization of ripe pistachio hull essential oil followed by the evaluation of antioxidant, cytoprotective, and antiperoxidative properties through cell-free and cell-based assays. The antimicrobial activity against some Gram-positive and Gram-negative standard and clinical bacterial has also been investigated.

## 2. Results and Discussion

### 2.1. Essential Oil Composition

The yield of hull essential oil of *Pistacia vera* L. variety Bronte (HEO) was 0.25% (*v*/*w* fresh material), well above others reported in literature (>3.50 times) [20], demonstrating how the Bronte variety possesses a much higher content of essential oil, probably due to characteristic pedoclimatic conditions of growth and probably to the extraction carried out immediately after harvest, which allowed for an increase in the extraction efficiency. This practice also led to a reduced formation of peroxidation, isomerization, or rearrangement of products due to temperature, light, and oxygen availability [29].

The essential oil composition, with retention indices and percentages, was reported in Table 1.

A total of 40 volatile constituents were identified, fully characterized, and grouped in four classes: monoterpene hydrocarbons, oxygenated monoterpenes, sesquiterpenes, and others (non-terpenoidic compounds). The major components of HEO were 4-Carene (31.743%), α-Pinene (23.584%), d-Limonene (8.002%), and 3-Carene (7.731%), all monoterpenes hydrocarbons that represented 86.591% of the HEO, followed by oxygenated monoterpenes, which presented an average value of 8.235% with α-Terpineol (4.036%), Borneol (0.831%), and α-Pinene oxide (0.787%) as the main compounds.

Although the monoterpenes hydrocarbons remained the most abundant family, followed by oxygenated monoterpenes (reflecting the results reported in the literature [20]), the characteristic absence of oxygenated sesquiterpenes and the very low presence of non-oxygenated sesquiterpenes, makes the essential oil of Bronte pistachio hulls easily recognized and distinguishable from essential oils derived from other pistachio hull varieties.

### 2.2. Antioxidant Activities

**Figure 1:**
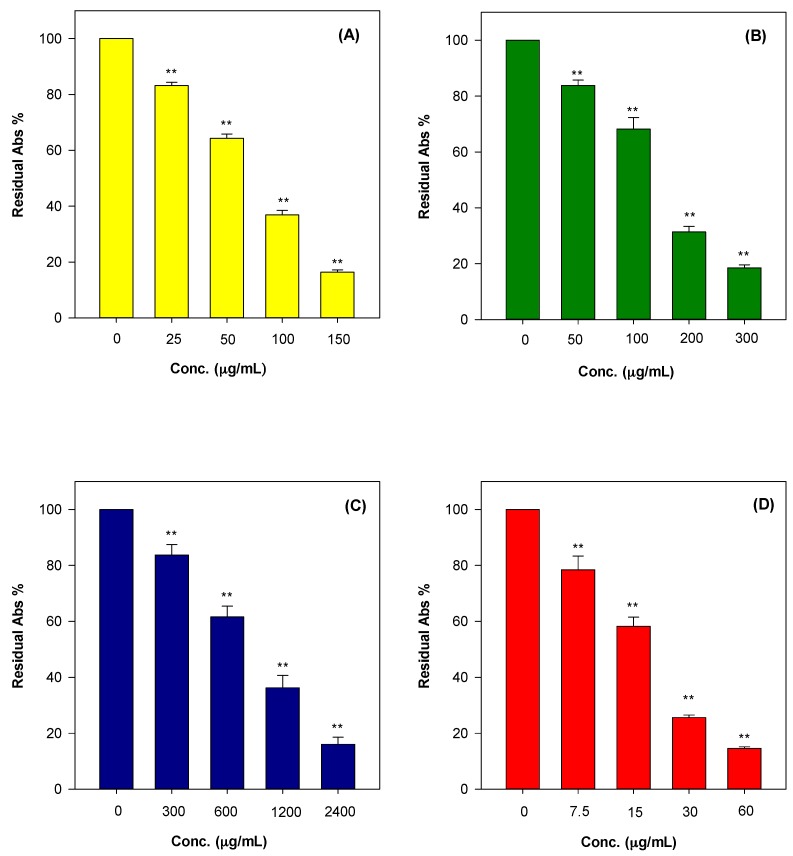
Antioxidant and free radical-scavenging activity of hull essential oil of *Pistacia vera* L. variety Bronte (HEO) towards Fe^3+^-TPTZ (**A**); ABTS^•+^ (**B**); DPPH^•^ (**C**); and iron chelating capacity (**D**). The asterisks (**) indicate significant differences (*p* < 0.05).

**Figure 2:**
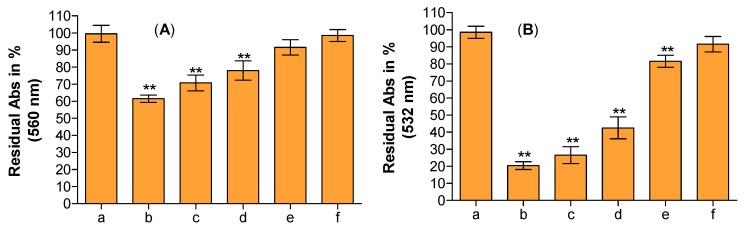
Superoxide anion (**A**) scavenging assay in the absence (a) or in the presence of 60, 30, 15, 7.5, and 3.7 μg/mL of HEO (b–f, respectively); Hydroxyl radical (**B**) scavenging assay in the absence (a) or in the presence of 15, 7.50, 3.75, 1.85, and 0.92 μg/mL of HEO (b–f, respectively). The asterisks (**) indicate significant differences (*p* < 0.05). Each value represents mean ± SD (*n* = 3).

Reactive oxygen species are involved in the pathological development of many important human diseases, thus antioxidants play a crucial role in human health due to their unquestionable beneficial effects on living organisms that enable them to overcome oxidative injuries, and modulate biological pathways and membrane functionality [23]. In this article, the authors conducted the analysis of antioxidant and free radical scavenging properties of HEO by using several antioxidant assays (hydrogen atom transfer and electron transfer-based methods) in order to check their behaviour under different reaction environments and mechanism typologies. This aspect is often overlooked but is critical in order to establish the structure-scavenging activity relationship of the major components of the phytocomplex investigated [24]. Figure 1 shows the antioxidant and free radical-scavenging potential of HEO towards Fe^3+^-TPTZ (A), ABTS^•+^ (B), and 2,2-diphenyl-1-picrylhydrazyl (DPPH)^•^ (C) as well as its iron chelating capacity (D). Moreover, the antiradical properties of HEO against two of the most common and dangerous reactive oxygen species produced in the organisms, superoxide anion (O_2_^•−^) and hydroxyl radical (^•^OH), was investigated and reported in Figure 2A,B. As can be seen in Figure 1 and Figure 2, the HEO showed a remarkable dose-dependent antioxidant and free-radical scavenging activity towards all assays performed, with the following order of potency, expressed as the half maximal inhibitory concentration (IC_50_), ^•^OH (IC_50_ 0.003 mg/mL) > Ferric Reducing Antioxidant Power (FRAP; IC_50_ 0.063 mg/mL) > Trolox Equivalent Antioxidant Capacity (TEAC; IC_50_ 0.128 mg/mL) > DPPH (IC_50_ 0.878 mg/mL) as well as having showed a strong iron chelating capacity (IC_50_ 0.017 mg/mL). The strong antioxidant activity was also confirmed by Folin-Ciocalteu assay results (1278.44 ± 41.79 mg gallic acid equivalents (GAE)/100 g of HEO).

**Figure 3:**
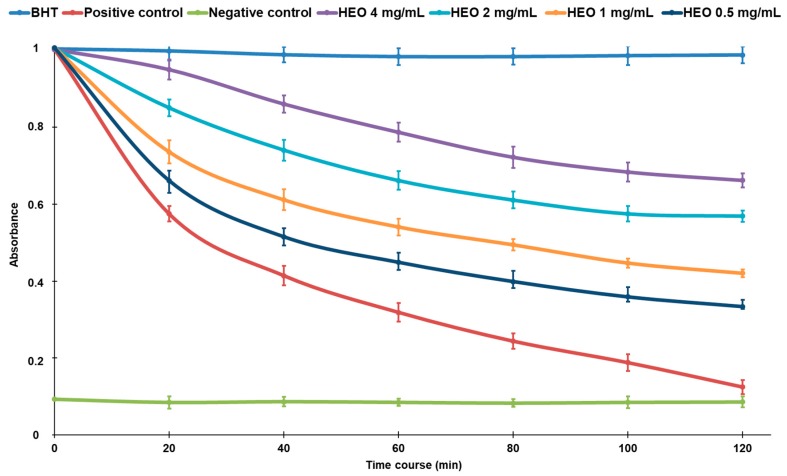
Beta-carotene bleaching curves of hull essential oil of *Pistacia vera* L. variety Bronte (HEO) at concentration range 0.5–4 mg/mL in respect to the reference compound butylated hydroxytoluene (BHT) (2 mg/mL). Results are expressed as mean (*n* = 3) of three independent experiments.

The ability to scavenge different reactive species makes the HEO an important source of antioxidants that are potentially useful in the detoxification mechanisms of living organism. Primary and relative weak antioxidants (such as O_2_^•−^), in fact, can be the precursor or be combined with others (e.g., nitric oxide) to give rise to very dangerous reactive species (hydrogen peroxide, hydroxyl radical, singlet oxygen, peroxynitric radical, and so on). Furthermore, HEO, acting as a strong chelating agent, may reduce the availability of transition metals and inhibit the radical-mediated oxidative chain reactions in biological systems preserving the integrity and consequently the functionality of membranes. As showed in β-carotene bleaching assay (Figure 3), in fact, the presence of these antioxidant compounds in HEO, mainly *p*-Cymene, Borneol, and β-Myrcene, are able to form radical adduct with peroxyl radical [30]; the compounds exhibit antioxidant properties and are capable of preventing oxidative damage from free radical-mediated oxidation in a dose-dependent manner.

These activities are predominantly attributable to the high amount of monoterpenes hydrocarbons found in HEO. In fact, it has widely been demonstrated that monoterpene hydrocarbons are better antioxidant compounds in respect to sesquiterpenes and particularly those with strongly activated methylene groups in their structure, such as 4-Carene, α-Terpinene, and γ-Terpinene, were the most active. So, in agreement with the data reported in literature [31], among oxygenated monoterpenes, the following order of effectiveness in antioxidant assays can be also hypothesised in our work: monoterpene phenols > allylic alcohols > monoterpenes aldehydes and ketones.

Concerning the sesquiterpene group, the radical scavenging properties of the hydrocarbons-type were quite low and lower than that of the monoterpene hydrocarbons group, whereas among the oxygenated type, mainly allylic alcohols showed good scavenging properties, similar to those of oxygenated monoterpenes [31], but they are completely lacking in this essential oil.

However, being a very complex matrix, the antioxidant properties of the essential oil do not always depend on the antioxidant activity of its main components and can be modulated by several mechanisms, so concepts of synergism and antagonism can be very relevant [30].

### 2.3. Cytoprotective Activity

**Figure 4:**
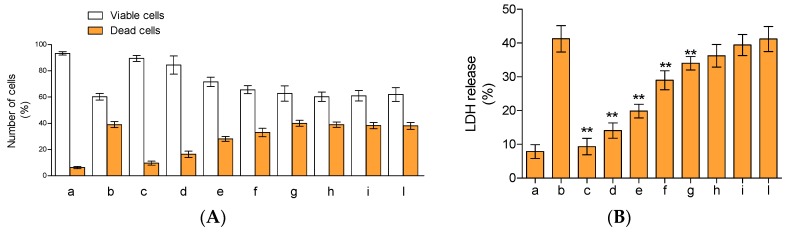
Cytoprotective effects of HEO on tert-butyl hydroperoxide (*t*-BOOH) treated lymphocytes. Lymphocytes plus 100 μM of *t*-BOOH were incubated for 24 h in the absence (b) or in the presence of 20, 17.5, 15, 12.5, 10, 7.5, 5, or 1 μg/mL of essential oil (c–l, respectively). Lymphocytes incubated under the same experimental condition without *t*-BOOH (a). Cell vitality and integrity were analysed by trypan blue staining (**A**) and lactate dehydrogenase (LDH) release (**B**). The asterisks (**) indicate significant differences (*p* < 0.05). Each value represents mean ± SD (*n* = 3).

The potential protective influences of the compounds present in HEO were analysed on lymphocytes treated with tert-butyl hydroperoxide (*t*-BOOH). A preliminary evaluation, obtained by incubating the cells with the essential oil at the concentration utilized in our work, shows no cytotoxicity effects of lymphocytes (data not shown). As can be seen in Figure 4, the treatment of lymphocytes with *t*-BOOH resulted in a net increase of cell mortality (~41% fall in cell viability vs. controls). The presence of 20, 17.5, 15, and 12.5 µg/mL of essential oil significantly increased cell viability by ~1.50, 1.40, 1.20, and 1.12-fold, respectively, compared cells treated only with *t*-BOOH (Figure 4A). Below this concentration, the effects were almost completely absent, with results superimposable with the ones obtained with lymphocytes treated with *t*-BOOH. The protective influences of the compounds present in the essential oil were also evident by monitoring lactate dehydrogenase (LDH) release in cell culture medium. In fact, this enzyme is commonly utilized as a marker of cell integrity and cytotoxicity, following oxidative burden, membrane damages, activation of apoptotic, and/or necrotic events. As we expected (Figure 4B), the incubation of lymphocytes with *t*-BOOH resulted in a remarkable increase of LDH release into the media (~4.2-fold higher than the control). The presence of 20 µg/mL of essential oil resulted in the complete absence of LDH release, confirming the data obtained with trypan blue coloration. The presence of 17.5, 15, 12, and 10 µg/mL of essential oil decreased enzyme release by ~66%, 52%, 31%, and 6%, respectively, in comparison to the sample treated with only *t*-BOOH, while concentrations below 10 µg/mL did not show a significant decrease of LDH release. Overall, the compounds present in the hull essential oil showed dose-response activity whereby they increased cell survival and prevented damages due to the presence of the strong antioxidant.

### 2.4. Antimicrobial Activities

The minimal inhibitory concentrations (MICs) and minimal bactericidal concentrations (MBCs) values of HEO against the American Type Culture Collection (ATCC) and clinical isolates tested bacteria are reported in Table 2. Results of negative controls indicated the complete absence of inhibition of all the strains tested (data not shown). A concentration of 7.11 mg/mL inhibited the growth of all the tested strains with the exception of *Pseudomonas aeruginosa* ATCC 9027. The same concentration was found to be bactericidal against all strains. Several studies have previously demonstrated the antimicrobial activity of plant essential oils [32,33,34]. The compound 4-carene, also identified amongst the main constituents of the Iranian Cymbobogon Olivieri essential oil, has been implicated in the antimicrobial activity against Gram-positive bacteria, Gram-negative bacteria, and the yeast *Candida albicans* [35].

The seed oil from the Tunisian endemic *Ferula tunetana* Pomel ex Batt. containing α-pinene (39.8%) was also found active against *Salmonella typhimurium* LT2 DT104 and *Bacillus cereus* ATCC 14,579, with inhibition zones of 16.2 ± 1.0 mm and 15.8 ± 1.0 mm, respectively [36]. Although we have not performed any mechanistic investigation in the present study, we believe the polyphenols present in HEO damaged the cell wall or cell membrane.

## 3. Materials and Methods

### 3.1. Chemicals

Folin-Ciocalteu reagent, 2,2-diphenyl-1-picrylhydrazyl (DPPH), potassium peroxydisulfate, 2,2′-azino-bis (3-ethylbenzothiazoline-6-sulfonic acid) diammonium salt (ABTS^•+^), 2,4,6-Tris(2-pyridyl)-*S*-triazina (TPTZ), ethylenediaminetetraacetic acid (EDTA), iron sulphate heptahydrate, ferrozine, potassium peroxydisulfate, sodium phosphate dibasic, potassium phosphate monobasic, sodium acetate, iron(III) chloride hexahydrate, iron(II) chloride tetrahydrate, and C7–C40 saturated alkane standard were purchased from Sigma-Aldrich (Milan, Italy). α-Pinene, 2-Carene, 3-Carene, β-Myrcene, and d-limonene were purchased from Extrasynthese (Lyon, France). Dichloromethane was GC-grade and was purchased from Merck (Darmstadt, Germany). All other chemicals and solvents used were of analytical grade.

### 3.2. Plant Material and Isolation of Essential Oil

The ripe pistachio hulls (*P. vera* L., variety Bronte) were harvested on 3 October 2016 by a local farmer in Bronte (Catania, Italy) and immediately sent to the laboratory. Four hundred grams (400 g) of fresh pistachio hulls were manually separated from the inner woody shells and subjected to hydrodistillation, according to the current *European Pharmacopoeia* method, until no significant increase in the volume of the collected oil was observed (3 h). The HEOs were dried on Na_2_SO_4_ and stored in a sealed vial under N_2_ until analysis.

### 3.3. GC/MS Analysis

Gas chromatographic (GC) analysis was performed on Agilent gas chromatograph, Model 7890A, equipped with a flame ionization detector (FID). Analytical conditions were HP-5MS capillary column (30 m × 0.25 mm coated with 5% phenyl methyl silicone, 95% dimethyl polysiloxane, 0.25 µm film thickness) and helium as the carrier gas (1 mL/min). Injection was done in split mode (1:60), injected volume was 1 µL (10% essential oil/CH_2_Cl_2_ *v*/*v*), and the injector and detector temperatures were 250 °C and 280 °C, respectively. The oven temperature was held at 50 °C for 1 min, then increased to 240 °C at 5 °C/min and held at 240 °C for 5 min. Percentages of compounds were determined from their peak areas in the GC-FID profiles.

Gas chromatography-mass spectrometry (GC-MS) analysis was carried out on the same instrument described above coupled with an Agilent 5975C mass detector (Santa Clara, CA, USA), with the same column and the same operative conditions used for the analytical GC. We adjusted the ionization voltage to 70 eV, the electron multiplier to 900 V, and the ion source temperature to 230 °C. Mass spectra data were acquired in the scan mode in an *m*/*z* range of 40–300. The compounds were identified based on: their GC retention index (relative to C9–C22 *n*-alkanes on the HP-5MS column), values reported in the literature, the computer matching of their mass spectral data with those of the MS library (NIST 08), the comparison of their MS fragmentation patterns with those reported in literature, and, whenever possible, the co-injection with authentic standards (α-Pinene, 2-Carene, 3-Carene, β-Myrcene and d-limonene).

### 3.4. Screening of Antioxidant and Free-Radical Scavenging Properties

#### 3.4.1. Determination of Total Phenolic Compounds

The total phenols content was determined according to Smeriglio et al. [37] using gallic acid as the reference compound and the results were expressed as mg of gallic acid equivalents (GAE)/100 g of essential oil.

#### 3.4.2. DPPH Assay

The DPPH free radical scavenging activity was evaluated according to Bellocco et al. [24]. Briefly, freshly DPPH methanol solution (10^−4^ M), was mixed with 37.5 μL of sample solution (range 2.4–0.300 mg/mL), and the mixture was vortexed for 10 s at room temperature (RT). The decrease in absorption at 517 nm, against blank, was measured after 20 min using an UV-VIS Spectrophotometer (Shimadzu UV-1601, Kyoto, Japan). The inhibition (%) of radical scavenging activity was calculated by the following equation:

(1) $$\mathrm{Inhibition}\;(\%)=\frac{A_{0}-A_{S}}{A_{0}}\times 100$$

where *A*_0_ is absorbance of the control and *A_S_* is absorbance of the sample after 20 min incubation.

#### 3.4.3. Trolox Equivalent Antioxidant Capacity (TEAC) Assay

The antioxidant activity against ABTS^•+^ radical was carried out according to Smeriglio et al. [37]. Briefly, the reaction mixture (4.3 mM potassium persulfate and 1.7 mM ABTS solution 1:5, *v*/*v*) was incubated for 12–16 h in the dark at room temperature, and was diluted, before use, with phosphate buffer (pH 7.4) in order to obtain an absorbance at 734 nm of 0.7 ± 0.02. Fifty microliters (50 µL) of sample solution (range 300–50 µg/mL) was added to 1 mL of reaction mixture and incubated in the dark at room temperature for 6 min; the absorbance was then recorded at 734 nm using an UV-VIS Spectrophotometer (Shimadzu UV-1601). The inhibition (%) of radical scavenging activity was calculated using Equation (1).

#### 3.4.4. Ferric Reducing Antioxidant Power (FRAP)

The free-radical scavenging capacity against TPTZ radical was performed according to Smeriglio et al. [37]. Twenty-five microliters (25 µL) of sample solution (range 150–25 µg/mL) was added to 1.5 mL of daily fresh FRAP reagent pre-warmed at 37 °C, and the absorbance was recorded at 593 nm by an UV-VIS Spectrophotometer (Shimadzu UV-1601) after an incubation time of 4 min at 20 °C, with the FRAP reagent used as blank. The inhibition (%) of radical scavenging activity was calculated using Equation (1).

#### 3.4.5. Chelating Capacity on Fe^2+^

Fe^2+^ chelating capacity was evaluated as described by Smeriglio et al. [37] with some modifications. Briefly, 25 µL of FeCl_2_∙4H_2_O solution (1.8 mM) was added to 50 µL of sample solution (range 60–7.5 µg/mL) and incubated at RT for 5 min. Following, 50 µL of a ferrozine solution (4 mM) was added to the reaction mixture, and the sample volume was diluted to 1.5 mL with deionized water. After that, the mixture was vortexed and incubated for 10 min at room temperature. The absorbance was read at 562 nm using an UV/VIS Spectrophotometer (Shimadzu UV-1601). The inhibition (%) of Fe^2+^ chelating capacity was calculated using Equation (1).

#### 3.4.6. β-Carotene Bleaching

The β-carotene bleaching assay was performed using an emulsion prepared according to Smeriglio et al. [37]. Aliquots of this emulsion (7.0 mL) were mixed with 0.28 mL of sample solution (range 4–0.5 mg/mL). An emulsion without β-carotene was used as control. The reaction mixture was initially recorded at the starting time (*t* = 0) at 470 nm and then incubated at 50 °C in a water bath for 120 min, with the absorbance recorded every 20 min. Butylated hydroxytoluene (BHT) was used as the reference compound and the results were expressed as inhibition (%) of β-carotene bleaching using Equation (1).

#### 3.4.7. Superoxide Anion (O_2_^•−^) Scavenging Assay

The superoxide anion scavenging activity of the sample was determined according to Barreca et al. [23]. The reaction mix was composed of 1.0 mL of nitroblue tetrazolium (NBT) solution (156 μM NBT in 100 mM phosphate buffer, pH 7.4), 1.0 mL of β-nicotinamide adenine dinucleotide (NADH) solution (468 μM in 100 mM phosphate buffer, pH 7.4), and 20 µL of sample solution (range 60–3.75 µg/mL). The reaction was started by adding 100 μL phenazine methosulphate (PMS) solution (60 μM PMS in 100 mM phosphate buffer, pH 7.4) to the mixture. The reaction mixture was incubated at 25 °C for 5 min, and absorbance at 560 nm was measured against blank samples with a Varian Cary 50 UV-VIS spectrophotometer. The inhibition (%) of radical scavenging activity was calculated using Equation (1).

#### 3.4.8. Hydroxyl Radical (^•^OH) Scavenging Assay

The hydroxyl radical scavenging assay was performed as described by Tellone et al. [38]. This assay is based on quantification of the degradation product of 2-deoxyribose by condensation with thiobarbituric acid (TBA). The reaction mixture contained the following in the final volume of 1.0 mL: 2.8 mM 2-deoxy-2-ribose, 10 mM phosphate buffer pH 7.4, 25 µM FeCl_3_, 100 µM EDTA, 2.8 mM H_2_O_2_, 100 µM ascorbic acid and sample solution in order to obtain the final concentration range of 15–0.92 µg/mL. The samples were incubated for 1 h at 37 ± 0.5 °C in a water bath. Then 1.0 mL of 1% (*w*/*v*) TBA was added to each mixture followed by the addition of 1 mL of 2.8% (*w*/*v*) trichloroacetic acid (TCA). The solutions were heated in a water bath at 100 °C for 15 min to develop the pink coloured malondialdehyde–thiobarbituric acid adduct. After cooling, the absorbance was measured at 532 nm against an appropriate blank solution. The inhibition (%) of radical scavenging activity was calculated using Equation (1).

### 3.5. Evaluation of Cytoprotective Properties

#### 3.5.1. Lymphocyte Isolation

Lymphocytes were isolated from heparinized whole blood collected from healthy volunteers, who provided written medical histories through a standardized questionnaire and had not taken anti-inflammatory medication or nutritional supplements. Blood samples were diluted with equal volumes of balanced salt solution, layered over Histopaque-1077 in centrifuge tubes and centrifuged at 400× *g* for 30–40 min at 25 °C. The peripheral blood mononuclear cell (PBMC) layer was removed with a pipette and washed by centrifugation. The PBMCs were passed through a Percoll gradient according to Repnik et al. [39] to enrich the fraction in lymphocytes. Lymphocytes (viability > 90%) were counted on a haemocytometer and suspended in Roswell Park Memorial Institute (RPMI) 1640 medium supplemented with 10% fetal calf serum, 2 mM glutamine, 100 units/mL penicillin G and streptomycin. Cell concentration was adjusted to 1 × 10^5^ cells/mL.

#### 3.5.2. Cytotoxicity Assays

For the cytotoxicity assay, cells (1 × 10^5^/mL) were incubated in complete medium without or with 20, 17.5, 15, 12.5, 10, 7.5, 5, or 1 µg/mL of sample solution for 24 h in the presence of 100 µM tert-butyl hydroperoxide (*t*-BOOH). The stock solution of HEO in dimethyl sulphoxide (DMSO) were conveniently diluted with phosphate saline buffer to maintain the concentration of the DMSO below 0.1% in the reaction mixture. Parallel controls were performed without *t*-BOOH and in the presence of the final concentrations of HEO. After incubation, cell viability was assessed by trypan blue staining. Briefly, an aliquot of the cell suspension was diluted 1:1 (*v*/*v*) with 0.4% trypan blue and the cells were counted using a haemocytometer. Results were expressed as the percentage of viable or dead cells (ratio of unstained or stained cells to the total number of cells, respectively). Cytotoxicity was also measured by lactate dehydrogenase (LDH) release from damaged cells into culture medium and expressed as a percentage of total cellular activity. LDH activity in the medium was determined using a commercially available kit from BioSystems S.A (Barcelona, Spain) and expressed as a function of the total amount of the enzyme present in the *t*-BOOH-no treated cells obtained after total cell lysis by sonication. At the concentration tested, HEO does not show any interference with the LDH assay.

### 3.6. Antimicrobial Activity

The following strains, obtained from the in-house culture collection of the University of Messina (Messina, Italy), were used for antimicrobial testing: *Staphylococcus aureus* ATCC 6538P, *Staphylococcus aureus* MRSA ATCC 43,300, three clinical isolates of *S. aureus* (of which two were methicillin resistant), *Escherichia coli* ATCC 10536, and *Pseudomonas aeruginosa* ATCC 9027.

Cultures for antimicrobial activity tests were grown in Mueller–Hinton Broth (MHB, Oxoid, CM0405) at 37 °C (24 h). For solid media, 1.5% (*w*/*v*) agar (Difco) was added.

The minimum inhibitory concentration (MIC) and the minimum bactericidal concentration (MBC) of HEO were determined by the broth microdilution method, according to CLSI (2009). MBCs were determined by seeding 20 mL from all clear MIC wells onto Mueller–Hinton agar (MHA, Oxoid) plates. The MBC was defined as the lowest extract concentration which killed 99.9% of the final inocula after 24 h incubation at 37 °C.

All experiments were performed in triplicate on three independent days. A number of positive and negative controls with selected antibiotics and solvent (DMSO) were included in each assay.

### 3.7. Statistical Analysis

Results (inhibition %) were expressed as mean ± standard deviation (S.D.) of three independent experiments in triplicate (*n* = 3) and as concentration inhibiting 50% of the initial activity (IC_50_). Data were analysed by one-way analysis of variance (ANOVA). The significance of the difference from the respective controls for each experimental test condition was assayed by using Tukey’s test for each paired experiment. Statistical significance was considered at *p* < 0.05. IC_50_ values were calculated by Graphpad Prism software (version 5.0, GraphPad Software Inc., La Jolla, CA, USA).

## 4. Conclusions

In the present study, we analysed, for the first time, the composition, as well as the antioxidant and biological potential of essential oil obtained from the hull of *Pistacia vera* L. variety Bronte. GC-MS analysis shed some light on its composition, allowing for the identification of 40 derivatives, characterized by the abundance of monoterpene hydrocarbons and oxygenated monoterpenes, both of which are some of the most studied and promising forms of secondary metabolites due to their remarkable and different biological properties. In fact, in the obtained results, the essential oil showed remarkable antioxidant and cytoprotective activities, which can also be attributed to its richness in monoterpene derivatives, which is dominated by the presence of 4-Carene, α-Pinene, d-Limonene, and 3-Carene. Therefore, this complex matrix could represent a suitable natural source of nutraceutical compounds to be employed in cosmetics, pharmaceutics, food preservation, and biotechnology applications.

## Figures and Tables

**Table 1:** Chemical composition of *Pistacia vera* L. variety Bronte hull essential oil.

#	KI ^a^	Compound	Area ^b^ (%)
1	916	Bornylene	0.035
2	923	Tricyclene	0.709
3	935	α-Pinene	23.584
4	950	Camphene	4.133
5	978	β-Pinene	1.062
6	993	β-Myrcene	2.393
7	995	2-Carene	1.152
8	1006	α-Phellandrene	0.456
9	1011	δ-3-Carene	7.731
10	1018	α-Terpinene	2.195
11	1027	*p*-Cymene	1.621
12	1031	d-Limonene	8.002
13	1050	*trans-*β-Ocimene	0.509
14	1056	*cis-*β-Ocimene	0.412
15	1061	γ-Terpinene	0.582
16	1082	4-Carene	31.743
17	1096	α-Pinene oxide	0.787
18	1101	Linalol	0.278
19	1107	2-Fenchanol	0.385
20	1130	1,3,8-*p*-Menthatriene	0.225
21	1148	Camphor	0.236
22	1150	Menthone	0.031
23	1169	Borneol	0.831
24	1188	*p*-Cymen-8-ol	0.692
25	1194	α-Terpineol	4.036
26	1197	Myrtenal	0.011
27	1202	Myrtenol	0.082
28	1210	α-Methylcynnamaldehyde	0.016
29	1250	Piperitone	0.687
30	1232	Nerol	0.272
31	1285	Bornyl acetate	2.430
32	1365	Nerol acetate	0.136
33	1513	β-Bisabolene	0.010
34	1525	γ-Selinene	0.005
35	1530	δ-Cadinene	0.028
36	1568	*cis*-5-Dodecenoic acid	0.225
37	1810	1,13-Tetradecadiene	1.528
38	1880	1-Hexadecanol	0.160
39	1957	Palmitic acid	0.015
40	2549	1,15-Hexadecadiene	0.430
41	2106	Unknown	0.013
42	2111	Unknown	0.129
Monoterpene Hydrocarbons			86.591
Oxygenated Monoterpenes			8.235
Sesquiterpenes			0.056
Others			5.115

**#**: Components are listed in their elution order from HP-5 MS column; ^a^: Retention index (KI) relative to standard mixture of *n*-alkanes on HP-5MS column; ^b^: values (relative peak area percentage) represent averages of three determinations.

**Table 2:** Minimal inhibitory concentrations (MICs) and minimal bactericidal concentrations (MBCs) of HEO (expressed as mg/mL) against Gram-positive and Gram-negative bacteria.

Bacterial Strain	MIC	MBC
*S. aureus* ATCC 6538	7.11	7.11
*E. coli* ATCC 10,536	7.11	7.11
*P. aeruginosa* ATCC 9027	na	na
*S. aureus* ATCC 43,300 (MRSA)	7.11	7.11
*S. aureus* 74CCH	7.11	7.11
*S. aureus* 7786	7.11	7.11
*S. aureus* 815	7.11	7.11

HEO, hull essential oil; na, not active.
